# COVID-19 monitoring of school personnel through molecular salivary test and dried blood spot analysis

**DOI:** 10.7189/jogh.14.05004

**Published:** 2024-02-09

**Authors:** Dolaji Henin, Clara Fappani, Daniela Carmagnola, Maria Gori, Gaia Pellegrini, Daniela Colzani, Antonella Amendola, Mariachiara Perrotta, Elisabetta Tanzi, Claudia Dellavia

**Affiliations:** 1 Department of Biomedical, Surgical and Dental Sciences, Università degli Studi di Milano, Milan, Italy; 2 Department of Health Sciences, Università degli Studi di Milano, Milan, Italy; 3 Coordinate Research Centre EpiSoMI (Epidemiology and Molecular Surveillance of Infections), Università degli Studi di Milano, Milan, Italy; 4 Department of Clinical Sciences and Community Health, Università degli Studi di Milano, Milan, Italy; 5 Coordinate Research Centre MACH (Centre for Multidisciplinary Research in Health Sciences), Università degli Studi di Milano, Milan, Italy

## Abstract

**Background:**

When the coronavirus disease 2019 (COVID-19) pandemic broke out, most countries enforced school closures as a precautionary measure. Although COVID-19 is still present three years later, schools have been reopened. We aimed to test the association of molecular salivary testing (MST) and dried blood spot (DBS) analysis for community surveillance by investigating the immunological profile of a group of school staff during and following COVID-19 vaccination.

**Methods:**

We conducted the study in a school in Milan from April 2021, when school staff were administered the first dose of vaccine against SARS-CoV-2, until the school year ended in June 2022. Each participant provided samples for MST and DBS one month (T1, W1) after receiving their first dose of vaccine. Subsequently, they collected weekly MST samples for five weeks (W2-W6), plus a DBS sample in the last week (T2). Both samples were collected one (T3), four (T4), and seven months (T5) after the administration of the second vaccine dose in May 2021. A final DBS sample was collected one year (T6) after T3.

**Results:**

Sixty participants provided 327 MSTs and 251 DBSs. None of the MST samples tested positive for SARS-CoV-2 RNA during the study period. A total of 201 DBS samples tested positive for the IgG semiquantitative analysis. Negative samples were found only at T1 (20.45%) and T2 (7.32%). We observed borderline results at T1 (4.55%), T2 (7.32%), and T4 (2.70%). The anti-SARS-CoV-2 average antibody ratio increased after the second dose between T2 and T3, and the trend peaked after the third dose between T4 and T6. We performed an immunoenzymatic assay of antibodies against nucleocapsid protein on samples collected at T1 from five participants who reported having been infected before the study and from four subjects with an abnormal increase in the antibody values at T4. Two samples tested positive in the first group and two in the second one.

**Conclusions:**

Our findings show that MST and DBS could be effective tools in the active surveillance of school personnel and that schools could be considered safe settings in view of SARS-CoV-2 infection. Vaccines might have contributed to case and/or symptom reduction.

At the beginning of the coronavirus disease 2019 (COVID-19) pandemic, when infections started clustering in some areas and causing a sudden, unexpected number of deaths [1], most countries enforced school closures, among other non-pharmaceutical interventions (NPIs), as a precautionary approach to mitigating viral transmission [2]. Three years later, COVID-19 remains present worldwide, but now schools are open.

The severe acute respiratory syndrome coronavirus 2 (SARS-CoV-2) was soon identified as the microorganism responsible for COVID-19. The most severe forms of the disease were mostly observed in individuals with comorbidities, pre-existing medical risk factors, and of older age [3]. The paediatric population generally experienced only minor forms of the disease [4,5]. With time, the interaction between SARS-CoV-2 and the host changed, and severe conditions of COVID-19 seem to occur less frequently in the overall population [6]. Contributing factors to such changes might be epidemiological, biological, and behavioural.

Since the outbreak of the pandemic, the SARS-CoV-2 genetic code has continuously mutated, and different variants have emerged and circulated globally. Initially, the B.1 SARS-CoV-2 lineage [6] was almost entirely replaced by the Alpha (January 2021) and later by the Delta variant (June 2021) [6–9]. The Centers for Disease Control and Prevention (CDC) classified them as ‘variants being monitored’ (VBM) [9], as they were associated with severe disease or a high viral transmission rate. In January 2022, the Omicron variant rapidly replaced Delta globally and was classified as a ‘variant of concern’ (VOC) [9] due to its high transmissibility, even in previously infected or vaccinated subjects. However, the variants caused less severe forms of disease than the original virus, most likely due to the integration of pharmaceutical protocols [10] and the introduction of vaccines worldwide [11].

With time, different vaccines against SARS-CoV-2 were developed and vaccine campaigns were organised [12]. In Italy, the first available vaccine, Comirnaty, BNT162b2 (Pfizer, New York, New York, USA), was introduced on 21 December 2020 and was initially reserved for health workers, then extended to the population >80 years old, and eventually offered to the whole population. The Moderna (Cambridge, Massachusetts, USA) vaccine followed shortly (early January 2021), with the same targets.

Vaxzevria (AstraZeneca, Cambridge, UK) became available at the end of January 2021 and was initially meant for the population 18–55 years of age. However, after some potentially related thromboembolic events were reported in March, it was reserved for the population >60 years old and finally abandoned in the summer of 2021. The vaccine campaign for school staff began in Italy in early 2021, when the Vaxzevria vaccine was primarily reserved for 18–55-year-old subjects. The booster doses for this population were later often administered at a time when the Vaxzevria vaccine had already been replaced with by the Comirnaty and Moderna vaccines. While the evidence of the durability and protection level of the vaccines is still limited, Monforte et al. [13] reported that vaccinated patients hospitalised for COVID-19 experienced a less severe disease than non-vaccinated ones, who, in turn, had an approximately 2-fold risk of in-hospital death.

Schools in Italy were gradually being closed between March and June 2020 and were reopened after the summer break in September 2020. In late October 2020, concomitantly with a new COVID-19 wave, distance learning was introduced for older pupils (age >16) together with stay-at-home measures and other NPIs. This was extended to middle school pupils (age >12) a few days later. Until the end of January 2021, only children 0–12 years of age were allowed to be present at school. As the number of infections decreased from February 2021 onwards, all schools eventually opened and never closed again. From September 2020 to September 2022, when the schools were reopening, attendance was allowed only by wearing masks, with strict distancing and following symptoms management protocols issued by the Italian National Health Institute [14].

Doubts have arisen quite early on the efficacy of school closure for the prevention of SARS-CoV-2 transmission, especially due to the expected disadvantages related to the lack of proper education, community, care and socialisation for children [2]. With time, knowledge concerning the transmission pathways of COVID-19 within communities and the social consequences of school closures constantly grew. However, the impact of school attendance or closure on COVID-19 transmission is not yet clear, as measuring and isolating such impact from other NPIs is not straightforward [15]. Nevertheless, many scientific reports seem to agree that children do not act as an infection threat [16] and that schools do not act as an infection multiplier [17].

Surveillance procedures, including the assessment of antibody titres and SARS-CoV-2 infection, have been introduced since the start of the pandemic for early COVID-19 detection, to investigate the individual immunological response to disease and vaccines. Beyond traditional serology by enzyme-linked immunosorbent assays (ELISA) or chemiluminescence assays on venous blood samples [18], dried blood spot (DBS) processing and analysis on capillary blood samples has proven useful as a non-invasive self-sampling methodology for the detection of SARS-CoV-2 type G immunoglobulins (IgG) [19]. Amendola et al. [19] reported that DBS is an acceptable alternative to plasma/serum for SARS-CoV-2 IgG detection, describing a significant concordance between the two tests based on samples collected from 52 healthcare workers. Morley et al. [20] observed a 98.1% sensitivity and 100% specificity of DBS compared to matched serum samples.

Concerning SARS-CoV-2 infection surveillance, the gold standard test for virus detection is the molecular nasopharyngeal swab (NPS) [21]. However, the less invasive molecular salivary test (MST), which is based on molecular analysis of induced saliva sample, showed similar reliability [22]. Yokota et al. [23] reported that MSTs have a 92% sensitivity and a >99% specificity, comparable to NPSs. Wyllie et al. [24] found that MSTs are more sensitive to SARS-CoV-2 detection in COVID-19 patients than NPSs. In their systematic review, Caixeta et al. [25] reported an 89% sensitivity, 96% specificity, and 93% accuracy of MSTs. In a previous study, our group reported the results of a school surveillance programme covering 401 students and 12 teachers for the early detection of SARS-CoV-2 infection using MSTs; we detected five positive cases during the six-week study period before the introduction of vaccines [26].

MST and DBS also share other advantages, such as their non-invasive, friendly nature, sample self-collection capability, and logistical sustainability, which might facilitate their combined use to provide a versatile instrument for viral and antibody surveillance and screening in communities, such as schools [19,26].

To assess such possibility, we aimed to test the validity and efficacy of the association of MST and DBS for community surveillance by investigating the immunological profile of a group of school staff during and after the introduction and administration of COVID-19 vaccines.

## METHODS

### Study Design

A school in Milan agreed to participate in a school surveillance programme designed by our group. The school hosted 813 students attending primary to high school (6–19 years of age) and 124 school staff, including 103 teachers and 21 administrative staff and janitors. Our study population were the school staff; we excluded the students because vaccination was only mandatory for adults at the time.

After presenting the project to the school’s principal and staff a few weeks before its start, we enrolled participants aged 18–65 years who received at least one dose of Vaxzevria before the start of the study and agreed to participate in the entire study cycle. Participation was voluntary, and all participants signed informed consent. We received approval from the local ethical committee (UNIMI – Number 108/20, 17/11/2020) and carried out the study following the principles of the Declaration of Helsinki.

**Figure 1:**
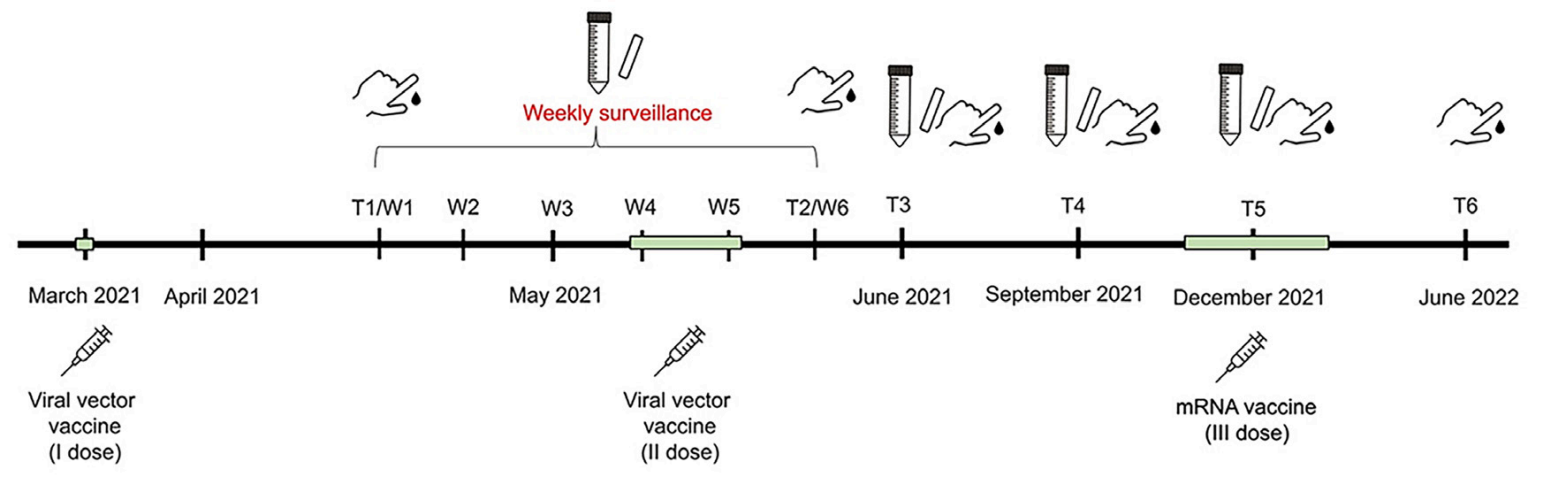
Timeline of anti-SARS-CoV-2 vaccinations and sample collection during the surveillance period, from March 2021 to June 2022.

The surveillance programme began in April 2021 after the administration of the first dose of a vaccine against SARS-CoV-2 and lasted until school closure in the second week of June 2022. Each staff member was asked to provide a saliva swab and a capillary blood sample (DBS) one month (T1, W1) after receiving their first vaccine dose in March 2021. Subsequently, they were asked to collect weekly saliva samples for five weeks (W2 to W6), plus a DBS sample in the last week (T2). New saliva and blood samples were then collected one (T3), four (T4), and seven (T5) months after the administration of the second vaccine dose in May 2021, followed by a final capillary blood sample one year (T6) after T3 (**Figure 1**).

The first part of the study (between the first and second vaccine doses) consisted of weekly MSTs aimed at rapidly detecting infected subjects, since a confident degree of immunity was still not reached, according to the Centers for Disease Control and Prevention (CDC) criteria [27]. As precise guidelines about immunological surveillance timing were never issued, we set the study timeline according to the school's logistical needs.

All participants filled out a questionnaire about their COVID-19 and vaccination history at the beginning of the study. Instructions for the self-collection of saliva for MSTs and blood for DBS analysis were attached to the questionnaire and were also delivered in a video emailed to each participant. They were required to fill in a second questionnaire about their updated COVID-19 history at T6.

### Samples collection

**Figure 2:**
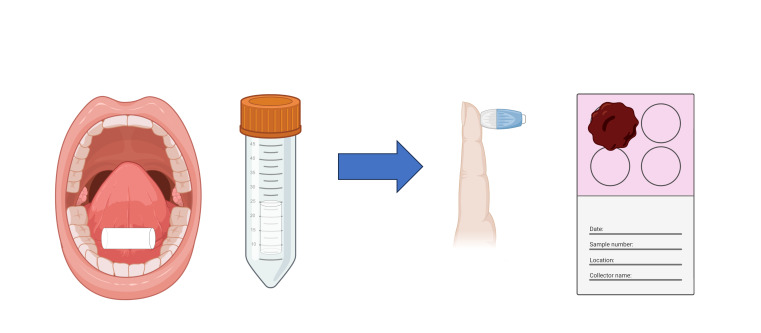
A representative figure of the sample collection. Created with BioRender.com.

Each participant received a pouch with a tube containing a dental roll for saliva collection and a Guthrie card with a lancing device for DBS collection. On a set day, saliva was self-collected in the morning at home by placing a dental roll in the mouth and holding it for three to four minutes in the lower vestibular space next to the premolar-molar area and under the tongue in the Wharton duct area. Once soaked, the rolls were put and preserved in a 50 mL sterile tube. DBS was self-obtained by pricking one forefinger with a lancet needle and collecting drops of blood on a specific Guthrie card in an adequate quantity to fill the contour of the card’s circle (**Figure 2**) [21,22,26].

The samples were placed back in the pouch and taken to school, where they were collected and delivered to our laboratory for analysis.

### Molecular investigation

We recovered saliva from the soaked dental roll by squeezing the roll employing a 10 mL disposable syringe. Samples were considered processable if at least 50 μL of saliva was recovered. Upon treatment with 7 μL of 800 U/mL proteinase K (New England BioLabs, Ipswich, MA, USA) and heat inactivation (5′ at 95°C), a 5 μL inactivated sample was tested for SARS-CoV-2 and RNase P detection by real-time reverse transcription polymerase chain reaction (real Time RT-PCR) according to the CDC diagnostic protocol [28]. Real-time RT-PCR assay was performed by TaqMan chemistry, using a Luna® Universal Probe One-Step RT-qPCR Kit (New England BioLabs, Ipswich, MA, USA) and The Applied Biosystem QuantStudio 5 Real Time PCR system (Thermo Fisher Scientific, Inc., Wilmington, DE, USA) [26]. Negative (nuclease-free water) and positive controls (2019-nCoV_N_Positive Control, IDT, Integrated DNA Technologies, Coralville, IA, USA) were included in each amplification round to validate the tests. We considered the results adequate if the presence of the RNase P gene was verified (cycle threshold (Ct)<35) and the samples were considered positive for SARS-CoV-2 if the N Ct growth curves crossed the threshold line within 40 cycles [26].

### Serological investigation

Capillary blood from each subject was collected on cellulose-based DBS cards (blood collection card, Euroimmun, Lübeck, Germany) through a finger prick. Samples were considered processable if at least one pre-printed spot was fully impregnated by blood. A 4.76 mm blood-impregnated disk was punched out of the card and incubated for one hour at 37°C in 250 μL of ELISA Sample Buffer (Euroimmun, Lübeck, Germany) to elute antibodies from the paper. According to the manufacturer's instructions, eluates were tested for anti-SARS-CoV-2 IgG antibodies using the semiquantitative anti-SARS-CoV-2 ELISA test (Euroimmun, Lübeck, Germany).

The results were interpreted semiquantitatively by a ratio calculated as the ratio of the sample's absorbance value to the calibrator's absorbance value (ODs/Cal). The ratio value of <0.8 was interpreted as negative, a ratio from ≥0.8 to <1.1 as borderline, and a ratio of ≥1.1 as positive.

Samples collected from subjects who provided at least three of the six requested ones were also tested using a quantitative anti-SARS-CoV-2 ELISA test. Elutes used for the semiquantitative analysis were further diluted using ELISA Sample Buffer according to the following ratio values: samples whose ratio value was 3.5 were tested as is; samples with a ratio value between 3.5 and 5 were diluted 1:10; and samples with a ratio value of 5 or higher were diluted 1:50. The diluted samples were then tested according to the manufacturer’s instructions. Results expressed as relative units (RU) per mL – were converted to binding antibody units (BAU) per mL using the conversion factor of 3.2 [19].

Antibody trends of subjects who had previously contracted SARS-CoV-2 infection or who showed unexpectedly high values not associated with recent vaccinations were further investigated through a semiquantitative analysis of anti-SARS-CoV-2 antibody against nucleocapsid protein (NCP) (ELISA-IgG, Euroimmun, Lübeck, Germany), according to the manufacturer’s instructions.

## RESULTS

### Subjects

Sixty staff members (7 males and 53 females, median age of 46 years, range: 24-64 years) agreed to participate in the study. This included 46 teachers and 14 administrative, technical, and auxiliary staff.

### Samples

During the study, 327 out of 540 expected saliva samples (60.56%) and 251 out of 360 expected DBS (69.72%) were collected (**Table 1**). Only 5 participants complied to collect a saliva sample at each set time point (8.33%), while 11 (18.33%) collected all the capillary blood samples required. An average of 5.5 saliva samples and 3.2 DBS were collected for each participant. The highest participation rate for saliva surveillance was observed at T1, W3, with 43 of the 60 expected samples (71.70%), while the highest compliance for the serological surveillance was observed at the T1, W1.

**Table 1:** The number of MST samples and DBS collected at each time point*

	T1 (W1)	W2	W3	W4	W5	T2 (W6)	T3	T4	T5	T6
**MST samples**	39	35	43	36	35	40	26	36	37	
**DBS**	55					53	33	39	37	34

DBS – dried blood spot, MST – molecular salivary testing, T – time point, W – week

*Empty cells indicate that samples were not collected.

Fifteen saliva samples were inadequate because of the low amount of saliva collected (15 out of 327 expected (4.59%)). The percentage of correctly collected samples increased as the study progressed, from 32 out of the expected 39 (82.10%) in the first week to all 35 out of the expected 35 (100.00%) at week 5. We observed a decline in December, with 34 eligible samples out of the expected 37 (91.90%).

Regarding DBS, 29 subjects out of an expected 360 (8.06%) did not collect enough blood for testing. The highest percentage of not properly collected samples was observed at T1 (n/N = 11/55 (20%)) and T2 (n/N = 12/53 (22.64%)) and decreased as the study progressed (3.03% at T3; 5.12% at T4; 8.12% at T5; and 0% at T6). A total of 222 DBSs was considered processable.

### COVID-19 history

All individuals were fully vaccinated (two doses) with the anti-COVID-19 viral vector vaccine: the first dose was administered in March 2021 and the second in mid-May 2021. Furthermore, 52 participants reported having received a booster dose (third dose) with an mRNA vaccine between December and January 2022, and 8 were not further vaccinated. None of the participants were infected during the weekly surveillance. Fifteen subjects contracted SARS-CoV-2 infection before the first immunisation cycle (before vaccination (BV)), while nine reported to have had COVID-19 after the third dose (after vaccination (AV)). Three of them had COVID-19 before the first and after the third dose. All AV cases happened from January 2022. In total, there were 21 subjects (24 episodes) who experienced COVID-19.

The BV infected subjects reported more severe symptoms than AV. BV reported fever, respiratory distress, and ageusia/ dysgeusia and symptoms’ duration ranged from 5 to 180 days with a mean of 34.37 days (standard deviation (SD) = 55.93). The AV group reported mild influenza-like symptoms with an average of 5.85 days of symptoms (SD = 3.27) (**Table 2**). Only one subject reported to have been infected at school.

**Table 2:** Symptoms declared by subject infected by SARS-CoV-2

			Infected twice	
**Symptoms**	**BV**	**AV**	**BV**	**AV**
Cold	3/12	4/6	2/3	3/3
Sinusitis	2/12	0/6	1/3	0/3
Fever	5/12	3/6	0/3	1/3
Anosmia/dysosmia	5/12	1/6	3/3	1/3
Ageusia/dysgeusia	5/12	2/6	3/3	1/3
Dyspnoea	3/12	0/6	0/3	0/3
Cough	4/12	2/6	0/3	0/3
Muscular and joint pain	1/12	1/6	0/3	1/3
Renal pain	1/12	0/6	0/3	0/3
Conjunctivitis	1/12	0/6	0/3	0/3
Lethargy	3/12	2/6	0/3	0/3
Headache	1/12	1/6	0/3	0/3
Gastro-intestinal symptoms	2/12	1/6	0/3	0/3

AV – after vaccination, BV – before vaccination

### Molecular surveillance

Despite being all adequate (RNase P positive), none of the 312 MST samples tested positive for SARS-CoV-2 RNA during the study period.

### Serological surveillance

A total of 201 DBS samples (201/222, 90.54%) tested positive to the IgG semiquantitative analysis. Negative samples were found only at T1, W1 (n/N = 9/44 (20.45%)) and T2 (6/41 (14.63%)). Borderline results were observed at T1 (n/N = 2/44 (4.55%)), T2 (n/N = 3/41 (7.32%)), and at T4 (n/N = 1/37 (2.70%)). Eleven subjects were negative or borderline one month after the administration of the first vaccine dose; two became positive before the second dose, six at one month after the second dose, and three failed to provide samples at T2 and T3.

**Figure 3:**
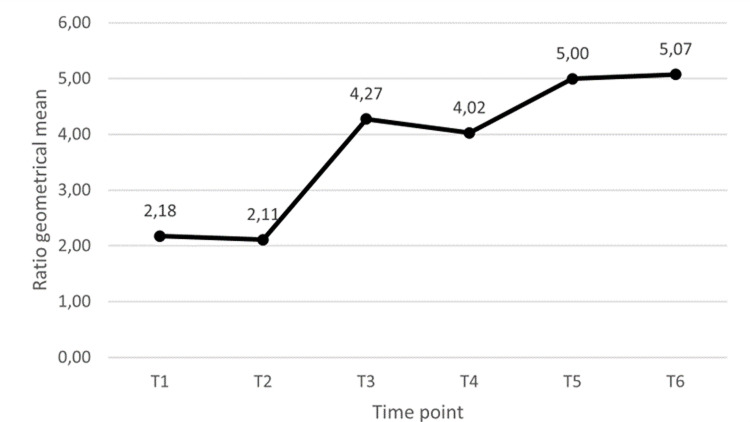
Anti SARS-CoV-2 antibody ratio trend represented as geometrical mean of ratio at each time point.

**Figure 4:**
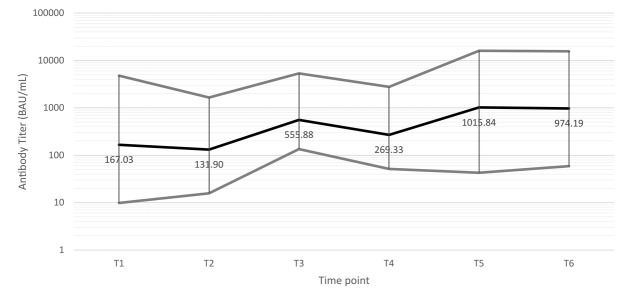
Trend of anti-SARS-CoV-2 antibody titres expressed as geometrical mean of single subject’s antibody titres at each time point (black line) and 95% confidence intervals (grey lines).

Regardless of the administration of the third dose, no samples collected at T6 tested negative. We also observed an increase in the antibody ratio in all subjects who had received the booster dose. **Figure 3** shows the geometric mean of the SARS-CoV-2 IgG ratio observed at each time point. We observed a similar trend considering the antibody titre of 33 participants who contributed to the project with 3 or more DBS during the study period (**Figure 4**).

Samples collected from five participants who reported having been infected with SARS-CoV-2 before the start of the study were tested for the presence of SARS-CoV-2 IgG antibody against NCP, indicative that an infection had occurred. Only two subjects showed the presence of these antibodies at T1 (**Table 3**). The same test was conducted on samples collected from four subjects who showed an abnormal increase in their antibody values three months after the administration of the second dose of vaccine (T4) (**Table 4**).

**Table 3:** Anti-SARS-CoV-2 antibodies against NCP protein ratio in five subjects with previous COVID-19 experience*

Subject ID	T1	T3	T4	T5
#1	0.4	0.3	0.3	0.4
#14	0.3		0.2	0.2
#17	1.3	1	1.1	
#21	1.2	0.8		0.5
#37	0.5		0.4	0.3

T – time point

*The ratio was evaluated in subjects who reported to have experienced SARS-CoV-2 infection before the administration of the first vaccine dose. Empty cells indicate that the sample was not collected.

**Table 4:** Anti-SARS-CoV-2 antibodies against NCP protein in subjects with abnormal increase in the antibody values*

Subject ID	Antibody test	T1	T2	T3	T4	T5	T6
#5	Anti-S (BAU/mL) anti-NCP (ratio)	27.552	16.32	280	828.8	2256	988.8
				2.1	1.3	1	
#9	Anti-S (BAU/mL)	4784	1664	1572.8	1943.2	841.6	1089.6
	Anti-NCP (ratio)			0.2	0.2	0.3	
#26	Anti-S (BAU/mL)	128.96		1444.8	5360	692.8	
	Anti-NCP (ratio)			0.2	0.2	0.2	
#45	Anti-S (BAU/mL)	2352	1600	792	1616		2128
	Anti-NCP (ratio)			3.1	2.1		

BAU – binding antibody unit, NCP – nucleocapsid protein, T – time point

*Values of anti-SARS-CoV-2 antibodies against NCP protein in four subjects who showed an abnormal increase in the antibody values three months after the administration of the second dose of vaccine (T4). Empty cells indicate that the sample was not provided or that the sample was not tested.

## DISCUSSION

The scientific community’s efforts to monitor SARS-CoV-2 circulation in the population through integrated molecular and serological surveillance proved to be crucial for understanding the dynamics, transmission mechanisms, and impact of COVID-19 in different groups of the population, but also for ensuring population safety. It has been estimated that 40.00% of SARS-CoV-2 positive cases are asymptomatic, and around 20.00% of transmission results from asymptomatic subjects, meaning that symptom screening alone is not sufficient for containing COVID-19 outbreaks [29,30].

The vaccination campaign contributed to decreasing the number of COVID-19 cases and deaths, limiting the transmission of the virus, and weakening symptoms [31]. Watson et al. [32] estimated that vaccinations prevented 14.4 million deaths in 185 countries and territories between December 2022 and December 2021.

Simultaneously, due to emerging SARS-CoV-2 variants, monitoring the immunological profile become key for formulating an effective long-term vaccination strategy, particularly in delicate communities such as school.

We proved MSTs and DBS samples might be performant in the school setting: The former for detecting the virus in the early stages of the infection and the latter for evaluating the antibody titre [26,33].

We therefore sought to follow the immunological profile of a small and homogeneous group of adults during and after they have been vaccinated, combining an already validated school surveillance protocol with MSTs and DBSs sampling. The rationale behind associating the two tests was to understand if an abnormal increase in the antibody titre could be related to a concomitant COVID-19 disease detected with MST. Furthermore, our previous school surveillance protocol was modified due to timing considerations: In the first part of the study, surveillance was conducted weekly in the time-lapse among the first and second dose to promptly detect positive COVID-19 cases, since full immunity has been estimated to reach its maximum only after the second dose in subjects with no experience of SARS-CoV-2 infection [34]. In the second part of the study, the lapse between each time point was extended to follow the antibody response of the participants during the school year. Therefore, we defined the time points at 1, 4, 7, and 13 months after the second dose, which was also in line with the study’s logistical needs. Before the start of the study, evidence regarding the antibody response dynamics was not robust, and existing literature reported only preliminary data [26,32–35]. Recent literature is still not unanimous in defining an adequate time-lapse for the evaluation of the antibody titre after vaccination. Aldridge et al. [35] conducted monthly surveys of a household community cohort of acute respiratory infections in England and Wales and obtained an antibody curve in subjects who had undergone different vaccines. Barbeau et al. [36] followed their sample population one, two, and six months post-initial vaccination. An observational study performed in Milan, Italy, surveyed 2179 healthcare workers for one year: two weeks, three months, six months, and one year after the second dose of the Comirnaty vaccine [37,38]. All the studies agreed to observe the change in antibody levels at least 14 days after the vaccine and to continue surveillance with wide time frames.

Concerning DBS results, 75.00% of the subjects showed serologic test positivity one month after the first dose and 100.00% one month after the second dose. An improvement of SARS-CoV-2 antibody positivity after the second dose of vaccine has been reported previously [39]. In our study, a slight decline in the antibody response observed at three months after the second dose, compared with the previous control (one month after second dose), was followed by an increase six months after the second dose, probably thanks to the fact that at least 43.00% of the participants had received their third dose. The booster dose administration effectively stimulated the immune system, since none of the triple immunised subjects showed an antibody decline at T5. The mean ratio at T5 and T6 was higher than that found at T2 (5 vs 4.27), which is in line with the findings by Firinu et al. [40], who observed higher anti-SARS-CoV-2 IgG levels following a heterologous administration of the booster dose than after two homologous vaccine administrations.

A quantitative ELISA test was performed on 33 subjects. The results of antibody titres in this subpopulation confirmed the mean trend of antibody response observed by semiquantitative analysis. However, the small population size and the lack of samples at each time point did not provide a complete picture of the participant's antibody curve. Moreover, the absence of sampling before the beginning of the vaccine administration (T0) did not allow us to evaluate the antibody response induced by the vaccine itself adequately.

Furthermore, the ELISA test against IgG directed toward NCP showed that only two subjects contracted SARS-CoV-2 infection during the surveyed period. NCP, being a specific structural component of SARS-CoV-2 unaffected by vaccination, is a suitable target for the indirect diagnosis of natural infection [19]. Such participants’ MSTs showed negative results for SARS-CoV-2 RNA at the time of the DBS sampling. Furthermore, the participants reported never having had COVID-19. It is therefore possible that the infection might have been active weeks before the collection of the MSTs and DBS samples and was asymptomatic. According to Amjadi et al. [41], IgGs directed toward the N protein peak three weeks after the onset of the symptoms/infection and can be detected for a month or more. Negative results to molecular testing could also be associated with a viral load lower than the limit of detection of the test (100.5 viral RNA copies/μL of sample) due to an early immune system response. This result also confirms that periodical surveillance might be more decisive than self-reported infections to exclude COVID-19 active cases [28].

All MSTs analysed were negative at all time points. The negativity of the swabs in T1-T2, T3, and T4 may be consistent with the fact that from April to September 2021 in Italy both new cases and hospitalisations decreased [42].

Furthermore, restriction measures such as the use of masks, green passes, and social distancing were applied in the school setting at this time. Even though Italy faced a new COVID-19 wave from September to December 2021, the tested subjects were COVID-19 negative. This suggests that schools were safe during the COVID-19 pandemic, considering that only adults were vaccinated [17].

The participants were asked through a questionnaire to report their COVID-19 experience at the beginning and the end of the study. To their knowledge, none reported having been infected in the first surveillance period, from April to May 2021. In fact, COVID-19 incidence decreased by 50% from April to June 2021 [43–45]. However, 9 subjects out of 60 reported to have been infected after vaccination, between February and May 2022. The COVID-19 positivity in this period could most likely be related to the contagiousness of the new variants overcoming the neutralising power of the antibodies generated during previous infections or vaccination [28,42]. Among these subjects, only one participant reported to be infected at school. Yan et al. [46] demonstrated that, during the highly transmissible Omicron wave, risk of infection in teacher-to-teacher contact was higher compared to student-to-student or student-to/from-teacher contact.

Omicron, differently from Alpha and Delta variants, has a high affinity for the upper respiratory tract, with symptoms such as sore throat, rhinorrhoea, and sneezing being reported frequently. It has been reported to cause less severe disease, hospitalisations, and risk of death [47]. The subjects enrolled in our study, infected after the vaccination in the January 2022 wave, experienced mild cold-like symptoms. This mildness, however, could also be related to the fact that we enrolled only non-immunocompromised subjects in the study, since those with pathologies affecting the immune system were not allowed to go to work in crowded spaces.

In our experience the school setting was characterised by a low risk for SARS-CoV-2 infection, since only one subject reported being infected at school. Several studies conducted in primary and secondary schools reported a low incidence of cases, monitoring both students and school personnel [17,46,48,49]. Macartney et al. [50] observed few cases and secondary infection rates among pupils and school personnel in a population of 8.1 million Australians. An active national surveillance programme performed in England found a low risk of infection in schools [51].

Our surveillance programme has several benefits, as already mentioned [17,52]: The self-collection of samples by patients is easy to perform and to accept, and can also reduce the need for direct interaction between healthcare workers and patients, as well as the risk of infection of the health personnel performing the testing. The sensitivity of molecular tests from a salivary swab is comparable to that of a NPS; moreover, SARS-CoV-2 RNA seems to be detectable in saliva earlier than in NPS when, at the level of the nasal mucosa, the viral load is still low, allowing early detection of the infection [53,54]. Regarding DBS sampling, it can help overcome logistics of the serological test, such as the sample collection, transport, and storage, with 96% of overall concordance with the quantitative test [19].

Nevertheless, only 8.00% and 18.00% of the participants provided MSTs and DBS samples at each time point, respectively. Furthermore, some MSTs and DBS samples were dry or inadequate. This suggests researchers should try to promote the participants’ compliance and improve collection instructions. Notably, as we performed our study between two academic years, some teachers were assigned to other schools and might have missed the samples delivery. Further, remote teaching was allowed during a part of the study period, and some teachers had no chance to provide the samples, as they taught from home.

Our study has some limitations, such as the low adherence and imperfect compliance of some participants. A side aim of projects like ours should be to build awareness in the populations they target, stressing the importance of adhering to community studies, while providing careful instructions and regular reminders. Furthermore, the study design and sample collection timeline were also influenced by pandemic-related factors, such as the onset of new variants, the introduction of different therapies and vaccines, therapies, and stay-at-home measures. Future studies in similar settings should anticipate and control for these factors in advance.

## CONCLUSIONS

The joint efforts to protect the population, develop new vaccines, and provide reliable therapies during the COVID-19 pandemic were outstanding. During the COVID-19 emergency, schools were closed as a precautionary measure. Should a new emergency occur in the future, we might learn from this one to take such decisions with more care. Building robust evidence to support the benefits or minimise the disadvantages of some decisions as quickly as possible is crucial during emergencies. In this respect, developing reliable and friendly tools, procedures, and instruments to screen the population, like the ones described here, may be useful to better evaluate possible restrictions and prevent their consequences.
